# Pancreatic disease patients are at higher risk for *Clostridium difficile* infection compared to those with other co-morbidities

**DOI:** 10.1186/s13099-019-0300-2

**Published:** 2019-04-23

**Authors:** Chetana Vaishnavi, Pramod K. Gupta, Megha Sharma, Rakesh Kochhar

**Affiliations:** 1 Department of Gastroenterology, Postgraduate Institute of Medical Education and Research, Chandigarh, 160012 India; 2 Department of Biostatistics, Postgraduate Institute of Medical Education and Research, Chandigarh, 160012 India

**Keywords:** Association, *Clostridium difficile* infection, Pancreatic disease, Renal disease, Hepatic disease, Malignancies

## Abstract

**Background:**

Surveillance of *Clostridium difficile* infection (CDI) in patients with underlying diseases is important because use of prophylactic antibiotics makes them prone to CDI. Epidemiology of CDI in this high-risk population is poorly understood. A study was conducted to evaluate the impact of CDI in patients with specific underlying co-morbidities.

**Method:**

A total of 2036 patients, whose fecal samples were processed for *C. difficile* toxin A and B assay by ELISA formed the basis of study. Patients with underlying diseases were classified based on the organ/kind of disease as pancreatic (*n *= 340), renal (*n *= 408), hepatic (*n *= 245), malignant (*n *= 517) and miscellaneous disease (*n *= 526). Laboratory records of clinical and demographic details were reviewed. The association of CDI with age, gender, antibiotic receipt, clinical symptoms and underlying co-morbidities was analyzed. Variation in CDI cases based on age groups was also investigated.

**Result:**

*Clostridium difficile* toxin positivity was 21.6% in general, whereas it was 30.6% in the pancreatic, 17.9% in the renal, 19.6%, in the hepatic, 21.3% in the malignancy and 20.0% in the miscellaneous disease groups. Toxin positivity was the lowest (14.8%) for female gender under renal disease and the highest (31.8%) for patients aged 40 to < 60 years, under pancreatic disease. Bloody diarrhea was a significant predictor for *C. difficile* toxin positivity. *C. difficile* toxin status irrespective to the underlying diseases was neither dependent on gender, age-groups or the number of antibiotics used. Association between patients’ gender, age and antibiotics receipt with underlying disease conditions, respective to *C. difficile* toxin status showed significance in relation to male gender (p < 0.05), age 40 to < 60 years (p = 0.03) and those receiving single (p = 0.09) or multiple antibiotics (p = 0.07).

**Conclusion:**

Pancreatic disease patients are at a higher risk for developing CDI, and particularly male gender, age 40 to < 60 years and those receiving antibiotics are at significant risk.

## Introduction

*Clostridium difficile* is the causative microbe for almost all cases of pseudomembranous colitis and 15–25% of antibiotic associated diarrhea [1]. In recent years, *C. difficile* infection (CDI) has been increasing in occurrence and severity leading to considerable morbidity and mortality in hospitalized patients [2]. *C. difficile* produces two potent toxins (A and B), which are responsible for the pathogenicity of the disease. Common clinical symptoms are fever, abdominal cramping, diarrhea with increased fecal leukocytes and resultant dehydration. CDI is a mounting public health challenge due to acquisition of the organism both nosocomially [3] and from the community [4]. Vaishnavi [5] has reviewed the established and potential risk factors for CDI, which include patients with concomitant diseases.

*Clostridium difficile* infection is believed to be predominantly due to the broad-spectrum use of antimicrobials. Patients with underlying diseases generally receive prophylactic antibiotics, making them prone to acquire CDI. The epidemiology of CDI in this high-risk population is poorly understood. Due to global increase, the surveillance of CDI precipitated by underlying diseases is important as there is very little literature investigating the same. Early detection of patients with high CDI risk, particularly those with comorbidities, might help in the appropriate clinical management of the disease. In a recent study, the association of CDI in patients with inflammatory bowel diseases (IBD) was investigated to assess the role of IBD as a risk factor [6]. However, contrary to expectations, IBD was not found to be a risk factor for CDI in our setting. As a further extension to the study, a retrospective, observational investigation was conducted to evaluate the association of CDI in patients with specific underlying co-morbidities like pancreatic, hepatic, renal and malignant diseases and compared with those of other miscellaneous diseases.

## Methods

As this study was based on secondary data recorded in the laboratory on pre-printed proformae, informed consent from patients was not required. This project was cleared ethically by the Institute Ethical Committee, which operates according to the Declaration of Helsinki.

### Patient population

This 2100 bedded tertiary care hospital is associated with premier medical institute of the country, known for medical education and research. This hospital caters to patients from large regions of North India inclusive of Chandigarh, Punjab, Haryana, Himachal Pradesh, Jammu and Kashmir, and some parts of Uttar Pradesh and Rajasthan. Consecutive patients, whose fecal samples were received with specific request by the clinicians for *C. difficile* toxin assay, formed the basis of investigation. Samples were received in the Microbiology Division of the Department of Gastroenterology from October 2009 to September 2016. Fresh samples were processed daily as a matter of routine for CDI diagnostic purposes. However patients with IBD were analyzed earlier [6] and therefore excluded from the present study.

### Inclusion and exclusion criteria

Consecutive patients of age group more than 2 years and with different underlying diseases, except IBD were included in the study. Patients less than 2 years and pregnant women were excluded from the study.

### Underlying disease categorization

During analysis, the patients were divided into the following groups based on their co-morbidities:

i. Pancreatic disease patients: This group comprised of 340 patients with pancreatic diseases, excluding pancreatic malignancy.
ii. Renal disease patients: This group comprised of 408 patients with all kinds of renal diseases, including post-renal transplants. Renal malignancies were excluded from this group.
iii. Hepatic disease patients: In this group 245 patients with all kinds of liver diseases except liver malignancies were included.
iv. Malignancy group patients: A total of 517 patients with all kinds of malignancies inclusive of hematologic, pancreatic, renal and liver malignancies were included in this group.
v. Miscellaneous disease patients: This group integrated 526 consecutive patients sent by the clinicians for *C. difficile* toxin investigation. None of the patients in this group had IBD or any of the above mentioned co-morbidities.

Laboratory data of all the included eligible patients using pre-designed specific data-format, maintained in the Department, were reviewed. The primary and secondary outcome was based on the *C. difficile* toxin status of the patient. Clinical symptoms suggestive of CDI, such as watery diarrhea, bloody diarrhea, presence of mucus in stool, abdominal pain, fever, frequency and duration of diarrhea were analyzed. Patient demographics, pertinent clinical aspects, diagnosis, therapy, antibiotic exposure and hospitalizations were taken into consideration along with the data of fecal *C. difficile* toxin A and B assay carried out as described earlier [7] using ELISA kits (DRG-International Inc, USA). Briefly, break away microtiter wells were coated with monoclonal anti-toxin A and polyclonal anti-toxin B antibodies directed against *C. difficile* toxins A and B respectively. An aliquot of fecal suspension was added to the wells and incubated; the non-bound material was removed by washing the wells three times with wash buffer. Another incubation was carried out with biotinylated polyclonal anti-toxin A and monoclonal anti-toxin B antibodies. Non-bound material was again removed by a washing step. During the next incubation period horseradish peroxidase-conjugated streptavidin was added which reacted with the bound biotinylated antibodies. Unbound conjugate was removed by a washing step and the reaction was terminated by sulfuric acid dispensed into the wells. The intensity of the developed color, which was directly proportional to the specifically bound amount of *C. difficile* toxin A and B, was measured in an ELISA reader (Tecan Infinite F50, Austria) at 450 nm. After consideration of the cut-off value, results were interpreted as positive or negative.

### Statistical analysis

The data were entered in MS Excel 2007 on 32-bit Microsoft Window Operating system and analysis was performed on R-Gui Version 3.4 statistical software on the same machine. Kruskal–Wallis and Chi-square test were employed for comparative analysis of the different groups. The Chi-square statistical test was based on p-value less than or equal to 0.05 criteria. Distributions of *C. difficile* toxin status with underlying disease were shown by “*n*” and percent (%) and their association was tested using Chi-square test statistics. When required, post hoc analysis based on Chi-square test was also performed. The distribution of underlying co-morbidities were summarized and similarly tested. If the association between the above said cases turned out to be significant, then association profiling was done by stratification of the data based on patients’ characteristics, antibiotic used, and clinical symptoms. Association of CDI was obtained in percent (%).

## Results

During the study period of 7 years, 307,299 patients were admitted to the various wards of the hospital. Of these stool samples from patients suspected to have CDI by the treating team were sent to our laboratory for *C. difficile* toxin assay. The study retrospectively looked if specific underlying diseases had any bearing on *C. difficile* toxin positivity. We thus accessed 2036 patients’ record data scrutinized on the basis of eligibility criteria. CDI was positive in 440 (21.6%) of the 2036 samples tested. Statistically adequate sample power was provided by this large number of patients available and even disease-wise the sample size is ≥ 245. Estimation of sample size was also done considering the association of 50%, 10% error and 95% CI and adjusted for design effect. The estimated sample size thus obtained for this extreme condition is 192. Therapies received by the patients were also reviewed.

### Pancreatic disease patients

Of the 340 pancreatic disease patients analyzed in the study, 232 (68.2%) were males and 108 (31.8%) females. The age range of the patients was 10–85 years (mean ± SD: 41 ± 14). There were 327 (96.2%) hospitalized patients, 6 (1.8%) outpatients and 7 (2.0%) patients with hospitalization status unknown. Major antibiotics received by these patients were nitroimidazoles (*n *= 107), penicillins (*n *= 88), carbapenems (*n *= 87), fluoroquinolones (*n *= 24), cephalosporins (*n *= 22), polymyxins (*n *= 11), aminoglycosides (*n *= 10), oxazolidinones (*n *= 8) and lincosamides (*n *= 6). Antifungals and proton pump inhibitors (PPI) were received by 5 each of the patients. However no patient received any form of immunosuppressant drugs or steroid treatment.

### Hepatic disease patients

In the patients with liver diseases (*n *= 245; M:F 188:57) the age ranged from 9 to 83 years (mean ± SD: 45 ± 14). There were 233 (95%) hospitalized patients, 6 (2.5%) outpatients and 6 (2.5%) patients whose hospitalization status was not known. Major antibiotics received by these patients were penicillins (*n *= 35), cephalosporins (*n *= 19), nitroimidazoles (*n *= 17), polymyxins (*n *= 11), carbapenems (*n *= 10), fluoroquinolones (*n *= 9), oxazolidinones (*n *= 2) and lincosamides (*n *= 2). Ten patients received PPI and 4 received immunosuppressants. But no patient received any form of steroid treatment.

### Renal disease patients

In the patients with renal disorders (*n *= 408; M:F 280:128) the age ranged from 10 to 90 years (mean ± SD: 42 ± 16). There were 318 (77.9%) hospitalized patients, 73 (17.9%) outpatients and 17 (4.2%) patients with unknown hospitalization status. Major antibiotic received by these patients were penicillins (*n *= 107), nitroimidazoles (*n *= 98), fluoroquinolones (*n *= 79), glycopeptides (*n *= 44), cephalosporins (*n *= 39), carbapenems (*n *= 29), aminoglycosides (*n *= 8), lincosamides and polymyxins (*n *= 5 each) and oxazolidinones (*n *= 2). Patients receiving antifungals were 3, antivirals 2 and antiprotozoal 1. Immunosuppressants were received by 19 and steroid treatment by 9 of the patients.

### Malignancy group patients

In the patients with malignancies (*n *= 517; M:F 350:167) the age ranged from 3 to 86 years (mean ± SD: 34 ± 23). There were 487 (94.2%) hospitalized patients, 20 (3.9%) outpatients and 10 (1.9%) patients with hospitalization status unknown. Major antibiotics received by these patients were carbapenems (*n *= 42), cephalosporins (*n *= 38), glycopeptides (*n *= 24), penicillins (*n *= 22), nitroimidazoles (*n *= 16), fluoroquinolones and aminoglycosides (*n *= 13 each), polymyxins (*n *= 10), oxazolidinones and lincosamides (*n *= 2 each). Antifungals were received by 5, antiviral by 13, steroids by 7 and immunosuppressant by a lone patient.

### Miscellaneous disease patients

There were 526 patients (M:F 325:201) who did not have IBD or any other above grouped co-morbidities. The age of the patients ranged from 3 to 103 years (mean ± SD: 41 ± 19). There were 432 (82.2%) hospitalized patients, 77 (14.6%) out patients and 17 (3.2%) patients with hospitalization status unknown. Major antibiotic received by these patients were penicillins (*n *= 55), glycopeptides (*n *= 53), nitroimidazole (*n *= 51), cephalosporins (*n *= 49), fluoroquinolones (*n *= 26), carbapenems (*n *= 18), aminoglycosides (*n *= 16), lincosamides and polymyxins (*n *= 4) and oxazolidinones (*n *= 3). Other drugs received by the patients were antifungals (*n *= 8), antivirals (*n *= 16), antiprotozoals (*n *= 2), PPI (*n *= 45), steroids (*n *= 38) and immunosuppressants (*n *= 16).

### Association of *C. difficile* toxin status and underlying disease conditions

We have also performed the logistic regression analysis to cross check and the result is not different as explained through stratified tabular results. Logistic regression with *C. difficile* versus disease conditions provides p-value < 0.05 only for patients with pancreatic diseases. Whereas adjusted p-value using rest of variables have similar findings. The odd ratio and adjusted odd ratio are 1.77 (1.29, 2.42) and 1.91 (1.35, 2.67). In addition bloody diarrhea is also reported significant (p-value < 0.05) irrespective to underlying diseases condition.

Association between patients’ *C. difficile* toxin status and underlying disease conditions, irrespective to all observed factors is depicted in Table 1 and found to be significant (p < 0.05). Distribution of patients with underlying disease conditions highlighted that proportion of hepatic disease patients was the smallest (12.0%) while those with miscellaneous disease was the highest (25.9%). Chi-square p-value of < 0.05 explained that the underlying disease condition is a risk factor for *C. difficile* toxin status and further post hoc analysis showed that pancreatic disease group was significant (p < 0.05) in association with the other underlying disease conditions. To comprehend the variation based on age groups, the patients were divided into four groups i.e. (i) < 20 years (ii) 20 to < 40 years (iii) 40 to < 60 years (iv) 60 years and above. Association of *C. difficile* toxin status with gender, age groups and antibiotic receipt, irrespective to underlying disease conditions (Table 2) was not found to be significant (p > 0.05).

**Table 1:** Association between patients’ CDT status and underlying disease condition irrespective to all observed factors

Underlying diseases	Patients n (%)	CDT pos n (%)	CDT neg n (%)	Chi-square p-value
Pancreatic diseases	340 (16.7)	104 (30.6)	236 (69.4)	< 0.05*
Hepatic diseases	245 (12.0)	48 (19.6)	197 (80.4)	
Renal diseases	408 (20.0)	73 (17.9)	335 (82.1)	
Malignancies	517 (25.4)	110 (21.3)	407 (78.7)	
Miscellaneous diseases	526 (25.9)	105 (20)	421 (80)	
Total	2036 (100)	440 (21.6)	1596 (78.4)	

Post Hoc analysis* Adjusted p-value*	Pancreatic diseases	Hepatic diseases		< 0.05*
		Renal diseases		
		Malignancies		
		Miscellaneous diseases		
	Hepatic diseases	Renal diseases		0.71
		Miscellaneous diseases		0.92
	Malignancies	Hepatic diseases		0.71
		Renal diseases		0.42
		Miscellaneous diseases		0.71
	Miscellaneous diseases	Renal diseases		0.71

*CDT Clostridium difficile* toxins, *pos* positive, *neg* negative

*Significant p-value

**Table 2:** Association of CDT status with gender, age and antibiotic receipt, irrespective to underlying diseases conditions

Gender, age and antibiotics received	Patients n (%)	CDT neg n (%)	CDT pos n (%)	Chi-square p-value
Gender				
Male	1375 (67.5)	1073 (78.0)	302 (22.0)	0.617
Female	661 (32.5)	523 (79.1)	138 (20.9)	
Total	2036 (100)	1596 (78.4)	440 (21.6)	
Age groups in years				
2 to < 20	294 (14.4)	227 (14.2)	67 (15.2)	0.773
20 to < 40	668 (32.8)	522 (32.7)	146 (33.2)	
40 to < 60	729 (35.8)	580 (36.4)	149 (33.9)	
60 and above	345 (17)	267 (16.7)	78 (17.7)	
Total	2036 (100)	1596 (78.4)	440 (21.6)	
Antibiotics receipt				
Nil	437 (21.5)	352 (80.6)	85 (19.4)	0.461
Single	689 (33.8)	535 (77.6)	154 (22.4)	
Multiple	910 (44.7)	709 (77.9)	201 (22.1)	
Total	2036 (100)	1596 (78.4)	440 (21.6)	

*CDT Clostridium difficile* toxins, *pos* positive, *neg* negative

Association between patients’ clinical symptoms and *C. difficile* toxin status, irrespective of underlying diseases condition is presented in Table 3 and that between patients’ gender, age groups and antibiotics receipt with underlying disease conditions, irrespective to *C. difficile* toxin status in Table 4. Underlying disease conditions irrespective to *C. difficile* toxin status are highlighted in Table 5.

**Table 3:** Association between patients’ symptoms and CDT status, irrespective to underlying diseases condition

Clinical symptoms	Patients n (%)	CDT pos n (%)	CDT neg n (%)	Chi-square p-value	
Bloody diarrhea					
Absent	1912 (93.9)	1508 (94.5)	404 (91.8)	0.05*	
Present	124 (6.1)	88 (5.5)	36 (8.2)		
Total	2036 (100)	1596 (78.4%)	440 (21.6%)		
Watery diarrhea					
Absent	768 (37.7)	613 (38.4)	155 (35.2)	0.25	
Present	1268 (62.3)	983 (61.6)	285 (64.8)		
Total	2036 (100)	1596 (78.4%)	440 (21.6%)		
Mucus in stool					
Absent	1364 (67.0)	1065 (66.7)	299 (68)	0.67	
Present	672 (33.0)	531 (33.3)	141 (32)		
Total	2036 (100)	1596 (78.4%)	440 (21.6%)		
Abdominal pain					
Absent	1193 (58.6)	951 (59.6)	242 (55)	0.09	
Present	843 (41.4)	645 (40.4)	198 (45)		
Total	2036 (100)	1596 (78.4%)	440 (21.6%)		
Fever					
Absent	1174 (57.7)	922 (57.8)	252 (57.3)	0.90	
Present	862 (42.3)	674 (42.2)	188 (42.7)		
Total	2036 (100)	1596 (78.4%)	440 (21.6%)		

Frequency	Median (IQR)	6 (4–8)	6 (4–8)	Ranksum test	0.521
Duration	Median (IQR)	3 (2–7)	3 (2–6)		0.119

*CDT Clostridium difficile* toxins

* Significant

**Table 4:** Association between patients’ characteristics with underlying diseases condition, irrespective to CDT status (n = 2036)

Characteristics	Pancreatic diseases n (%)	Hepatic diseases n (%)	Renal diseases n (%)	Malignancies n (%)	Miscellaneous diseases n (%)	Chi-square p-value
Gender						
Male	232 (68.2)	188 (76.7)	280 (68.6)	350 (67.7)	325 (61.8)	< 0.05*
Female	108 (31.8)	57 (23.3)	128 (31.4)	167 (32.3)	201 (38.2)	
Total	340 (100)	245 (100)	408 (100)	517 (100)	526 (100)	
Age groups in years						
2 to < 20	12 (3.5)	5 (2)	24 (5.9)	184 (35.6)	69 (13.1)	< 0.05*
20 to < 40	157 (46.2)	73 (29.9)	157 (38.5)	98 (18.9)	183 (34.8)	
40 to < 60	129 (37.9)	128 (52.2)	164 (40.2)	143 (27.7)	165 (31.4)	
60 and above	42 (12.4)	39 (15.9)	63 (15.4)	92 (17.8)	109 (20.7)	
Total	340 (100)	245 (100)	408 (100)	517 (100)	526 (100)	
Antibiotics received						
Nil	50 (14.7)	36 (14.7)	117 (28.7)	80 (15.5)	154 (29.3)	< 0.05*
Single	123 (36.2)	111 (45.3)	104 (25.5)	153 (29.6)	198 (37.6)	
Multiple	167 (49.1)	98 (40)	187 (45.8)	284 (54.9)	174 (33.1)	
Total	340 (100)	245 (100)	408 (100)	517 (100)	526 (100)	

*CDT Clostridium difficile* toxins

* Significant p-value

**Table 5:** Association between patients’ clinical symptoms and underlying diseases condition, irrespective to CDT status

Clinical symptoms	Pancreatic diseases n = 340 (%)	Hepatic diseases n = 245 (%)	Renal diseases n = 408 (%)	Malignancies n = 517 (%)	Miscellaneous diseases n = 526 (%)	Chi-square p-value		
Bloody diarrhea								
Absent	324 (95.3)	236 (96.3)	389 (95.3)	484 (93.6)	479 (91.1)	0.01*		
Present	16 (4.7)	9 (3.7)	19 (4.7)	33 (6.4)	47 (8.9)			
Watery diarrhea								
Absent	113 (33.2)	100 (40.8)	144 (35.3)	207 (40)	204 (38.8)	0.17		
Present	227 (66.8)	145 (59.2)	264 (64.7)	310 (60)	322 (61.2)			
Mucus in stool								
Absent	190 (55.9)	165 (67.3)	299 (73.3)	355 (68.7)	355 (67.5)	< 0.05*		
Present	150 (44.1)	80 (32.7)	109 (26.7)	162 (31.3)	171 (32.5)			
Abdominal pain								
Absent	156 (45.9)	148 (60.4)	263 (64.5)	309 (59.8)	317 (60.3)	< 0.05*		
Present	184 (54.1)	97 (39.6)	145 (35.5)	208 (40.2)	209 (39.7)			
Fever								
Absent	172 (50.6)	171 (69.8)	277 (67.9)	240 (46.4)	314 (59.7)	< 0.05*		
Present	168 (49.4)	74 (30.2)	131 (32.1)	277 (53.6)	212 (40.3)			

Frequency	Median (IQR)	5 (4–8)	6 (5–8)	6 (4–8)	5 (4–8)	5 (4–8)	Kruskal–Wallis test	0.006*
Duration	Median (IQR)	3 (2–10)	3 (2–5)	3 (2–5)	3 (2–4.5)	5 (3–15)		< 0.001*

*CDT Clostridium difficile* toxins, *Pos* positive, *Neg* negative

* Significant p-value

Association between patients’ gender, age groups and antibiotic receipt with underlying disease conditions, respective to *C. difficile* toxin status (Table 6) showed significance in relation to male gender (p < 0.05), age 40 to < 60 years (p = 0.03) and receipt of single (p = 0.09) and multiple antibiotics (p = 0.07). But association between patients’ clinical symptoms and CDI respective to underlying diseases conditions (Table 7) was found to be non-significant (p > 0.05) in relation to clinical symptoms. The association of CDI was stratified based on the underlying disease conditions and further on gender, age-group and number of antibiotics used.

**Table 6:** Association between patients’ characteristics and CDT status infection respective to underlying diseases condition

Characteristics	No. of total patients	Pancreatic diseases	Hepatic diseases	Renal diseases	Malignancies	Miscellaneous diseases	Chi-square p-value
Gender							
Male							
CDT pos	302	74	37	54	71	66	< 0.05*
CDT neg	1073	158	151	226	279	259	
Total	1375	232	188	280	350	325	
Female							
CDT pos	138	30	11	19	39	39	0.23
CDT neg	523	78	46	109	128	162	
Total	661	108	57	128	167	201	
Age groups in years							
2 to < 20							
CDT pos	67	5	1	6	40	15	0.75
CDT neg	227	7	4	18	144	54	
Total	294	12	5	24	184	69	
20 to < 40							
CDT pos	149	36	25	24	32	32	0.13
CDT neg	581	93	103	141	111	133	
Total	730	129	128	165	143	165	
40 to < 60							
CDT pos	146	50	13	30	20	33	0.03*
CDT neg	521	107	60	126	78	150	
Total	667	157	73	156	98	183	
60 and above							
CDT pos	78	13	9	13	18	25	0.82
CDT neg	267	29	30	50	74	84	
Total	345	42	39	63	92	109	
Antibiotics receipt							
Nil							
CDT pos	85	13	8	19	16	29	0.80
CDT neg	352	37	28	98	64	125	
Total	437	50	36	117	80	154	
Single							
CDT pos	154	39	23	16	34	42	0.09
CDT neg	535	84	88	88	119	156	
Total	689	123	111	104	153	198	
Multiple							
CDT pos	201	52	17	38	60	34	0.07
CDT neg	709	115	81	149	224	140	
Total	910	167	98	187	284	174	

*CDT Clostridium difficile* toxins, *pos* positive, *neg* negative

* Significant p-value

**Table 7:** Association between patients’ symptoms and CDT status respective to underlying diseases condition (n = 2036)

Symptoms	Pancreatic diseases (%)	Hepatic diseases (%)	Renal diseases (%)	Malignancies (%)	Miscellaneous diseases (%)	p-value
Bloody diarrhea						
Pos	6	4	4	7	15	0.65
Neg	10	5	15	26	32	
Total	16	9	19	33	47	
Watery diarrhea						
Pos	69	30	50	65	71	0.06
Neg	158	115	214	245	251	
Total	227	145	264	310	322	
Mucus in stool						
Pos	42	17	20	31	31	0.29
Neg	108	63	89	131	140	
Total	150	80	109	162	171	
Abdominal pain						
Pos	55	28	27	49	39	0.07
Neg	129	69	118	159	170	
Total	184	97	145	208	209	
Fever						
Pos	49	17	23	58	41	0.18
Neg	119	57	108	219	171	
Total	168	74	131	277	212	

*CDT Clostridium difficile* toxins, *Pos* positive, *Neg* negative

## Discussion

*Clostridium difficile* is largely spread by the feco-oral route and it is believed that underlying disease is a risk factor for CDI development [5]. The reduction of risk factors upon exposure to microbes is important to control CDI [8]. Though there are several co-morbidities associated with CDI, the available studies are mostly related to IBD [6, 9, 10] malignancy [11–13] or solid organ transplantation [14, 15]. In the present study we evaluated CDI in patients with specific underlying co-morbidities like pancreatic, hepatic and renal diseases and patients with malignancies and compared them with patients having other miscellaneous conditions.

In general, male gender was found to be strongly associated with various underlying diseases compared to females. *C. difficile* toxin positivity was not found to be significantly associated with the clinical symptoms and with the use of antibiotics in all the underlying disease groups. In an earlier study involving 3044 patients with suspected CDI, Vaishnavi et al. [7] found that fever (41%) was the most significant clinical symptom present, followed by abdominal pain (37.9%) in *C. difficile* toxin positive cases and was highly associated with renal diseases (20.8%), hepatic disorders (18.5%) and cancers (17.6%). In the present study *C. difficile* toxin status irrespective to the underlying diseases was neither dependent on gender, age-group or the number of antibiotics used.

The association of CDI based on 2036 patients’ data was computed 21.6% which is not the correct representation because the distribution of CDI prevalence depends on the underlying co-morbidities of the patients. The association of CDI stratified based on the underlying disease condition and further based on gender, age-groups and the number of antibiotics used showed highest association (30.6%) in pancreatic disease group and lowest in the renal disease group (17.9%). It was thus clear that pancreatic disease condition is a risk factor for CDI as compared to other underlying diseases. Similar condition was noted for association of CDI for pancreatic disease group when stratified by patients’ clinical symptoms. Association between patients’ gender, age and antibiotics received with underlying diseases condition, respective to *C. difficile* toxin status showed significance in relation to male gender (p < 0.05), in age 40 to < 60 years (p = 0.03) and receipt of single (p = 0.09) and multiple antibiotics (p = 0.07).

*Clostridium difficile* infection is commonly reported as nosocomial [3] and community acquired [4] with 22% hospital acquired cases in liver transplant patients [16]. The Canadian Nosocomial Infection Surveillance Program reported that of 1430 cases 62 (4%) CDI patients had underlying hepatic disease [17]. Musa et al. [18] reported CDI to be significantly more common amongst cirrhotics with hepatorenal syndrome. Bajaj et al. [19] observed that CDI independently increased the mortality in cirrhotic hospitalized patients. In the present study, *C. difficile* toxin was positive in 19.6% of the hepatic patients and the use of antibiotics in this group was found to be highly significant compared to the control miscellaneous disease group.

Keven et al. [14] in a 4 year study period reported 39 (5.5%) CDI cases among 600 kidney and 102 pancreas–kidney allograft transplants, with the latter patients having a slightly higher incidence of CDI than recipients of kidney alone. Arrich et al. [20] described CDI in an 82 year old man with acute renal failure. Eui et al. [21] retrospectively (2004–2008) investigated 85 CDI patients and reported a highly significant difference in chronic kidney disease prevalence between CDI and non-CDI patients, suggesting that chronic kidney disease as an independent risk factor for CDI development. Several other workers have also reported that patients with chronic kidney diseases have a higher risk of CDI and rise in nosocomial morbidity and mortality [22, 23]. In the present study in patients with renal disease, *C. difficile* toxin was positive in 17.9% of them and the duration of diarrhea was also significant compared to other co-morbid groups, except the miscellaneous disease group which was similar to the renal group.

There is hardly any literature available relating CDI with pancreatic diseases. In the present study, *C. difficile* toxin positivity (30.6%) in the pancreatic disease group was found to be highly significant compared to all the other specific groups (malignancies, renal and hepatic diseases) as well as the control patients. The use of antibiotics was also found to be significant in the pancreatic disease patients compared to those in the renal disease group and the control miscellaneous disease patients.

Patients with hematological malignancies [11, 13, 24, 25], post-transplant [26] post-chemotherapy patients [27, 28] and those with solid cancers [15, 29] can be predominantly vulnerable to CDI. This is due to the presence of multiple risk factors for CDI, which include extended hospital stays, exposure to multiple antibiotics and repeated cycles of chemotherapy. Gastrointestinal mucosal damage occurs from conditioning regimen/radiation or graft-versus-host disease of the gastrointestinal tract [30], and serve as independent risk factors for the development of CDI [5, 31]. Receiving antibiotics in addition can further increase the risk of acquiring CDI. In the present study the use of antibiotics was found to be highly significant in all the underlying disease groups irrespective of *C. difficile* toxin positivity status.

Antibiotics have been established as a risk factor for development of CDI [5, 32]. Bajaj et al. [33] reported that in-patient antibiotic use was an independent predictor of CDI in cirrhotic patients. Daniel and Rapose [34] retrospectively analyzed 100 CDI patients in a community hospital and observed that patients who had taken antibiotics in the previous 6 months constituted 74% of the total study population. In the present study, the use of antibiotics was significant in all the groups with specific underlying diseases. Though we did not find that antibiotic use precipitated CDI, these findings imply that CDI must be ruled out in all diarrheic patients with underlying diseases, as underlying diseases can themselves precipitate CDI. Therefore, these patients should be treated aggressively before the infection becomes complicated.

Reduced gastric acid due to PPI use leads to survival of any ingested *C. difficile* [35–37]. Apart from this, PPIs may also suppress the immune response to infection [38]. One study evaluating the relationship between PPI use and CDI in hepatic disease patients revealed that outpatient PPI use was an independent risk factor for CDI [39]. Daniel and Rapose [34] reported that more than 50% patients were on PPIs at the time of admission among 100 CDI patients analyzed with co-morbidities including malignancy (28%), diabetes mellitus (25%) and chronic renal disease (23%). In the present study PPI was used by 45 of the miscellaneous disease patients, 10 of the hepatic patients and 5 of the pancreatic disease patients.

Immunosuppressant medication is often required in certain patients with underlying diseases and is an important risk factor for CDI [40]. In the present study immunosuppressants were used by 19 of the renal group patients, 16 of the miscellaneous disease group patients, 4 of the hepatic group patients and 1 of the malignancy group patients. Similarly, corticosteroid is also a significant risk factor for patients with underlying disease [41]. In the present study, 38 patients in the miscellaneous disease group, 9 in the renal group and 7 in the malignancy group received steroids. However, no patient in the pancreatic group or the hepatic group received any form of steroid treatment.

The high association of CDI thus reported in patients with various underlying diseases, particularly the pancreatic group followed by malignancy group, and the considerable rate of severe cases, signifies the requirement for precautionary policies, such as antimicrobial stewardship programs, strict compliance with hand hygiene and environmental decontamination particularly involving this patient group. But despite the routine steps being taken to curb infection with a Hospital Infection Control Committee to constantly check the compliance, actually no decrease in the cases of CDI has been noted. Various factors may account for this. Our institute is a tertiary care hospital catering to the people of the northern region of India, inclusive of Chandigarh, Punjab, Haryana, Himachal Pradesh, Jammu and Kashmir, western parts of Uttar Pradesh and some parts of Rajasthan. Thus patients are referred from other lower centers where implementation of infection surveillance is not available. These patients are on antibiotics previously by the time they reach our hospital. Because of uncontrolled use of antibiotics, the difficulty in controlling antibiotic resistance occurs, despite adequate care being taken in our hospital.

The strength of this study is that this is the first analysis of its kind investigating CDI in underlying disease patients. However there are some limitations of this study. Firstly, the use of ELISA has its own limitations in detecting the toxins, but this method is widely used the world over. Moreover the kits we used had sensitivity up to 98% and specificity up to 92% and the assay was performed by a dedicated trained medical technician and therefore was largely reliable. Though molecular tests can also detect and confirm cases, the use of polymerase chain reaction to detect toxin A or toxin B genes has a potential for false positive results, given its high sensitivity, as PCR will detect even low number of *C. difficile* organisms transiently present in other hospitalized individuals with no CDI, and thus lead to wrong CDI diagnosis.

Secondly, of all the admitted patients, we had access to only those referred to us for *C. difficile* toxin assay. If it was surveillance or screening for *C. difficile* toxin, then the total number of patients with different diseases would be important. But this would have also resulted in tremendous cost to the hospital, which was not feasible in a low budget country.

Thirdly, the cases were not classified according to severity as the data assessment was limited to laboratory details of the patients without access to details on further clinical complications. Thus we had no access to the mortality rate data also. Some data of the patients’ prescriptions could also have been lost due to some likely incomplete records. But, as it is a tertiary care hospital, every effort is routinely made to maintain the demographic and clinical records for future use. However, as this is a preliminary study, further study based on severity classification will be carried out for individual group of diseases.

## Conclusion

The study looked retrospectively if specific underlying diseases had any bearing on *C*. *difficile* toxin positivity. Among the underlying diseases, pancreatic disease patients are the most susceptible to CDI compared to those with non-pancreatic diseases in our setting. Male gender, age 40 to < 60 years and those patients receiving antibiotics were also more prone to CDI. However, further studies are required to investigate the association of CDI in underlying diseases within the groups analyzed due to their complex pathophysiology.
